# Genomic Analysis of Carbapenem-Resistant Acinetobacter baumannii Strains Recovered from Chilean Hospitals Reveals Lineages Specific to South America and Multiple Routes for Acquisition of Antibiotic Resistance Genes

**DOI:** 10.1128/spectrum.02463-22

**Published:** 2022-09-26

**Authors:** Barbara P. Brito, Jonathan Koong, Aniela Wozniak, Andres Opazo-Capurro, Joyce To, Patricia Garcia, Mehrad Hamidian

**Affiliations:** a Australian Institute for Microbiology and Infection, University of Technology Sydney, Ultimo, New South Wales, Australia; b Laboratory of Microbiology, Department of Clinical Laboratories, Escuela de Medicina, Pontificia Universidad Católica de Chile, Santiago, Chile; c Millennium Initiative for Collaborative Research on Bacterial Resistance (MICROB-R), Santiago, Chile; d Clinical Laboratories Network, Red de Salud UC-CHRISTUS, Santiago, Chile; e Laboratorio de Investigación en Agentes Antibacterianos, Departamento de Microbiología, Facultad de Ciencias Biológicas, Universidad de Concepción, Concepción, Chile; Brown University

**Keywords:** *Acinetobacter baumannii*, carbapenem resistance, ST1, ST15, ST79, ST109, ST162, antibiotic resistance, whole-genome sequence, South America, Chile, plasmids

## Abstract

Carbapenem-resistant Acinetobacter baumannii (CR*Ab*) is a public health threat accounting for a significant number of hospital-acquired infections. Despite the importance of this pathogen, there is scarce literature on A. baumannii molecular epidemiology and evolutionary pathways relevant to resistance emergence in South American strains. We analyzed the genomic context of 34 CR*Ab* isolates recovered from clinical samples between 2010 and 2013 from two hospitals in Santiago, Chile, using whole-genome sequencing. Several Institut Pasteur scheme sequence types (STs) were identified among the 34 genomes studied here, including ST1, ST15, ST79, ST162, and ST109. No ST2 (the most widespread sequence type) strain was detected. Chilean isolates were phylogenetically closely related, forming lineages specific to South America (e.g., ST1, ST79, and ST15). The genomic contexts of the resistance genes were diverse: while genes were present in a plasmid in ST15 strains, all genes were chromosomal in ST79 strains. Different variants of a small Rep_3 plasmid played a central role in the acquisition of the *oxa58* carbapenem and *aacC2* aminoglycoside resistance genes in ST1, ST15, and ST79 strains. The *aacC2* gene along with *bla*_TEM_ were found in a novel transposon named Tn*6925* here. Variants of Tn*7* were also found to play an important role in the acquisition of the *aadA1* and *dfrA1* genes. This work draws a detailed picture of the genetic context of antibiotic resistance genes in a set of carbapenem-resistant A. baumannii strains recovered from two Chilean hospitals and reveals a complex evolutionary picture of antibiotic resistance gene acquisition events via multiple routes involving several mobile genetic elements.

**IMPORTANCE** Treating infections caused by carbapenem-resistant A. baumannii (CR*Ab*) has become a global challenge given that CR*Ab* strains are also often resistant to a wide range of antibiotics. Analysis of whole-genome sequence data is now a standard approach for studying the genomic context of antibiotic resistance genes; however, genome sequence data from South American countries are scarce. Here, phylogenetic and genomic analyses of 34 CR*Ab* strains recovered from 2010 to 2013 from two Chilean hospitals revealed a complex picture leading to the generation of resistant lineages specific to South America. From these isolates, we characterized several mobile genetic elements, some of which are described for the first time. The genome sequences and analyses presented here further our understanding of the mechanisms leading to multiple-drug resistance, extensive drug resistance, and pandrug resistance phenotypes in South America. Therefore, this is a significant contribution to elucidating the global molecular epidemiology of CR*Ab*.

## INTRODUCTION

Nosocomial infections are associated with high mortality and morbidity rates worldwide, including in both developed and developing countries (1). Acinetobacter baumannii is a Gram-negative opportunistic pathogen often associated with hospital-acquired infections (i.e., ventilator-associated pneumonia and wound and blood infections), in which carbapenems such as imipenem and meropenem are considered “last-line” therapeutic options to treat serious infections caused by this microorganism (2). Carbapenem-resistant A. baumannii (CR*Ab*) isolates, which are also resistant to a wide range of other antibiotics, have been increasingly reported worldwide, representing a serious threat to public health (3, 4). The World Health Organization has deemed CR*Ab* a “critical-priority pathogen” for which new therapeutic options are urgently required (5).

Carbapenem resistance in A. baumannii is due mainly to the presence of horizontally acquired OXA-type carbapenem resistance genes, including *oxa23*, *oxa24*, and *oxa58*, in most geographical regions (6, 7). The presence of an insertion sequence (IS) (often IS*Aba1*) upstream of the intrinsic *oxaAb* gene (also known as *bla*_OXA-51-like_) has also been shown to be involved in carbapenem resistance in A. baumannii (8).

CR*Ab* outbreaks are normally associated with members of a limited number of globally distributed clonal complexes such as sequence type 2 (ST2), ST25, and ST85 (all STs refer to the Institut Pasteur scheme unless otherwise indicated) (6, 9–11). Interestingly, evidence has demonstrated that CR*Ab* isolates belonging to the major global clones (ST1 and ST2) are rarely found in South America, while other sequence types such as ST15 and ST79 are prevalent in this region (12–14). In Chile, A. baumannii was responsible for about 98% of cases of ventilator-associated pneumonia in hospitalized adults in 2018, with rates of susceptibility to imipenem and meropenem being 44.6% and 40.4%, respectively (15). This represents a significant decrease in susceptibility with respect to reports from previous years (15). Despite its relevance, published molecular epidemiological studies of CR*Ab* in Chile and South American countries are scarce (6). To date, CR*Ab* isolates belonging to ST15, and its single-locus variant ST318, have been identified as the predominant lineage in the country (12, 14). However, the genetic structures of regions containing antibiotic resistance genes and pathways leading to resistance have not been characterized. Here, we studied a set of 34 A. baumannii isolates recovered from infections in two Chilean hospitals between 2010 and 2013. This study provides a detailed overview of complex evolutionary pathways involving several known and novel transposons (Tns), plasmids, and genomic islands leading to the generation of resistant lineages specific to South America.

## RESULTS

### Antibiotic resistance profile and sequence type distribution.

We determined the susceptibilities to seven categories of antibiotics. All of the strains were MDR (multidrug-resistant) strains, which are considered nonsusceptible (intermediate or resistant) to at least one agent in at least three categories of antibiotics (16). XDR (extensively drug-resistant) strains are defined as being nonsusceptible to at least one agent in all but one or two categories. Eighty percent (27/34) of the strains were nonsusceptible to six out of the seven categories tested. Analysis of the complete genomes showed that 3 strains belong to ST1, 10 strains belong to the ST15/ST318 complex, 5 strains belong to ST79, 10 strains belong to ST109, and 6 strains belong to ST162. In addition to the draft assemblies, representatives from each of the STs were chosen, and their genomes were completed using a hybrid assembly approach (using the Illumina short-read and Nanopore long-read technologies).

### Tn*6925*, a novel composite transposon carrying *bla*_TEM_ and *aacC2* inserted into a small plasmid in isolates belonging to global clone 1 (ST1:ST231:KL1:OCL1).

Only three global clone 1 (GC1) strains were found (UC22850, UC22863, and UC23742) (Tables 1 and 2). All three strains were resistant to ampicillin-sulbactam, ceftazidime, cefoperazone-sulbactam, imipenem, meropenem, gentamicin, amikacin, and ciprofloxacin but remained susceptible to tigecycline and colistin (Table 1). Consistent with their phenotypes, all three genomes contained the *aphA6* amikacin, *sul1* sulfonamide, *catA1* chloramphenicol, *tetA* tetracycline, *bla*_TEM_ ampicillin, and *aacC2* gentamicin-tobramycin resistance genes. UC22850, UC22863, and UC23742 contained a copy of IS*Aba1* upstream of the chromosomal *ampC* and *oxaAb* genes (encoding OXA-69), accounting for their resistance to third-generation cephalosporins and carbapenems, respectively. Analysis of the UC22850 complete genome, chosen as the representative of GC1 strains (ST1 strains), showed that the *aphA6* amikacin resistance gene was in Tn*aphA6* in a novel chromosomal location (bases 1491184 to 1494255 in the sequence under GenBank accession number CP076821).

**TABLE 1:** General properties and antibiotic resistance profiles of the isolates studied

Strain	Year of isolation	Hospital-ward^*b*^	Source^*c*^	Resistance profile, MIC (mg/L)^*a*^									
				SAM	CAZ	CFP-SUL	IMI	MER	Ak	Gm	CIP	TGC	CST
UC22850	2011	A-1	Catheter	R, 32	R, >128	I, 32	R, 16	R, >16	R, 64	R, >16	R, >4	S, 2	S, 2
UC22863	2011	B	Blood	R, 32	R, >128	I, 32	R, 8	R, >16	R, 64	R, >16	R, >4	S, 0.5	S, 2
UC23742	2011	A-2	PT	R, 32	R, >128	I, 32	R, 8	R, >16	R, 64	R, >16	R, >4	S, 2	S, 2
UC20371	2010	C	Leg tissue	R, >32	R, >128	R, 64	R, 16	R, >16	R, 64	R, >16	R, >4	S, 2	S, 2
UC20932	2010	A-3	Leg wound	R, 32	R, >128	R, 64	R, >16	R, 16	R, 64	S, ≤4	R, >4	S, 2	S, 2
UC21698	2010	B	Leg tissue	S, ≤8	R, >128	S, 16	R, 8	R, 16	R, 64	S, ≤4	R, >4	S, 2	S, 2
UC22488	2010	B	ETA	R, >32	R, >128	I, 32	I, 4	R, 8	R, 64	R, >16	R, >4	S, 2	S, 2
UC22807	2011	C	Leg bone	S, ≤8	R, >128	S, 16	R, 16	R, >16	R, 64	S, ≤4	R, >4	S, 2	S, 2
UC24137	2011	C	SU	R, >32	R, >128	R, 64	R, >16	R, >16	R, 64	R, >16	R, >4	S, 2	S, 2
UC25816	2012	A-4	Blood	R, >32	R, >128	R, 64	R, 16	R, >16	R, 64	R, >16	R, >4	S, 2	S, 2
UC27639	2013	A-4	Urine	R, >32	R, >128	R, 64	I, 4	R, 8	R, 64	R, >16	R, >4	S, 2	S, 2
UC21460	2010	C	Tissue	R, 32	R, >128	I, 32	R, >16	R, >16	R, 64	S, ≤4	R, >4	S, 2	S, 2
UC23723	2011	B	Blood	R, 32	R, >128	S, 16	R, >16	R, >16	R, 64	S, ≤4	R, >4	S, 2	S, 2
UC23022	2011	C	Blood	R, 32	R, >128	I, 32	R, >16	R, >16	R, 64	R, 16	R, >4	S, 0.5	S, 2
UC25431	2012	A-5	IT	R, >32	R, >128	R, 64	R, >16	R, >16	R, 64	R, 16	R, >4	S, 1	S, 2
UC25532	2012	A-6	Blood	R, 32	R, >128	I, 32	R, >16	R, >16	R, 64	R, >16	R, >4	S, 2	S, 2
UC25534	2012	A-6	ETA	R, 32	R, >128	I, 32	R, >16	R, >16	R, 64	R, >16	R, >4	I, 4	S, 2
UC25565	2012	B	Leg tissue	R, 32	R, >128	I, 32	R, >16	R, >16	R, 64	R, 16	R, >4	S, 1	S, 2
UC20804	2010	A-7	PA	R, 32	R, >128	I, 32	R, >16	R, 16	R, 64	R, >16	R, >4	S, 2	S, 2
UC21421	2010	Outpatient	Leg wound	R, 32	R, >128	I, 32	R, >16	R, 16	S, 8	R, >16	R, >4	S, ≤2	S, 2
UC22612	2010	A-1	ETA	R, 32	R, >128	I, 32	R, >16	R, 16	R, 64	R, >16	R, >4	R	S, 2
UC23844	2011	Outpatient	Ankle wound	R, 32	R, >128	I, 32	R, >16	R, 8	S, 8	R, >16	R, >4	S, 2	S, 2
UC24803	2011	A-3	ETA	R, 32	R, >128	I, 32	R, >16	R, 8	R, 64	R, >16	R, >4	S, 2	S, 2
UC25167	2012	A-8	ETA	R, >32	R, >128	I, 32	R, >16	R, 16	R, 64	R, >16	R, >4	S, 2	S, 2
UC25224	2012	B	Blood	R, 32	R, >128	S, 16	R, >16	R, 16	R, 64	R, >16	R, >4	S, 2	S, 2
UC25235	2012	A-5	LW	R, >32	R, >128	I, 32	R, >16	R, 16	R, 64	R, >16	R, >4	S, 2	S, 2
UC25271	2012	A-8	Leg tissue	R, 32	R, >128	I, 32	R, >16	R, 16	R, 64	R, >16	R, >4	S, 2	S, 2
UC25560	2012	A-3	PL	R, 32	R, >128	I, 32	R, >16	R, 16	S, 16	R, >16	R, >4	S, 1	S, 2
UC20520	2010	B	BAL fluid	I, 16	R, >128	S, 16	R, >16	R, >16	R, 64	S, ≤4	R, >4	S, ≤2	S, 2
UC20976	2010	B	Urine	I, 16	R, >128	S, 16	S, 2	R, 8	R, 64	S, ≤4	R, >4	S, 0.25	S, 2
UC21311	2010	A-8	ETA	R, 32	R, >128	S, 16	R, >16	R, >16	R, 64	R, >16	R, >4	S	S, 2
UC21742	2010	A-9	Drain fluid	R, 32	R, >128	S, 16	R, >16	R, >16	R, 64	R, >16	R, >4	S, 2	S, 2
UC24371	2011	C	ETA	I, 16	R, >128	S, 16	R, >16	R, >16	R, 64	R, 16	R, >4	S, 2	S, 2
UC25604	2012	B	PL	R, 32	R, >128	S, 16	R, >16	R, >16	R, 64	R, 16	R, >4	S, 2	S, 2

a SAM, ampicillin-sulbactam; CAZ, ceftazidime; CFP-SUL, cefoperazone-sulbactam; IMI, imipenem; MER, meropenem; Gm, gentamicin; Ak, amikacin; CIP, ciprofloxacin; TGC, tigecycline; CST, colistin; R, resistant; I, intermediate; S, susceptible.

b Samples were collected from three different hospitals within the network, called A, B, and C here, with their wards indicated by numbers.

c PT, periprosthetic tissue; PL, periprosthetic liquid; PA, peritoneal abscess; ETA, endotracheal aspirate; LW, laparoscopic wound; IT, inguinal tissue; SU, sacrum ulcer; BAL, bronchoalveolar lavage.

**TABLE 2:** Genomic properties of isolates examined in this study

Strain	ST^IP^^*a*^	ST^OX^^*b*^	OC	KL	Antibiotic resistance gene(s)	GenBank accession no.
UC22850	1	231	1	1	*aphA6*, *sul1*, *tetA*(A), *catA1*, *aacC2*, *bla*_TEM_	CP076821, CP076822
UC22863	1	231	1	1	*aphA6*, *sul1*, *catA1*, *tetA*(A), *aacC2*, *bla*_TEM_	JAHIDA000000000
UC23742	1	231	1	1	*aphA6*, *sul1*, *catA1*, *tetA*(A), *aacC2*, *bla*_TEM_	JAHICZ000000000
UC20371	15	225	7	22	*aphA6*, *sul2*, *bla*_TEM_, *catA2*, *aacC2*, *bla*_TEM_	JAHICY000000000
UC20932	15	236	7	9	*aphA6*, *oxa58*	JAHICX000000000
UC21698	15	236	7	9	*aphA6*, *sul2*, *strAB*	JAHICW000000000
UC22488	15	225	7	22	*aphA6*, *aacC2*, *bla*_TEM_	JAHICV000000000
UC22807	15	225	7	22	*aphA6*, *sul2*, *aacC2*, *bla*_TEM-1B_, *catA2*, *aacC2*, *bla*_TEM_	JAHICU000000000
UC24137	15	225	7	22	*aphA6*, *sul2*, *aacC2*, *aadA*, *oxa23*, *aadB*, *bla*_TEM_, *floR*	CP076817–CP076820
UC25816	15	225	7	22	*aphA6*, *sul2*, *aacC2*, *bla*_TEM_	JAHICT000000000
UC27639	15	225	7	22	*aphA6*, *sul2*, *aacC2*, *bla*_TEM_, *catA*	JAHICS000000000
UC21460	318	225	7	22	*aphA6* (2×), *oxa58*	CP076814–CP076816
UC23723	318	225	7	22	*aphA6* (2×), *oxa23*	JAHICR000000000
UC23022	79	1283	1	9	*aphA6*, *sul2*, *aadA1*, *dfrA1*, *strAB*, *cmlB1*, *oxa23*, *bla*_TEM_	CP076812, CP076813
UC25431^*c*^	79	1283	1	9	*aphA6*, *sul2*, *aadA1*, *dfrA1*, *strAB*, *cmlB1*, *oxa23*, *bla*_TEM_	JAHICQ000000000
UC25532	79	1283	1	9	*aphA6*, *sul2*, *aadA1*, *dfrA1*, *strAB*, *cmlB1*, *oxa23*, *bla*_TEM_	JAHICP000000000
UC25534	79	1283	1	9	*aphA6*, *sul2*, *aadA1*, *dfrA1*, *strAB*, *cmlB1*, *oxa23*, *bla*_TEM_	JAHICO000000000
UC25565^*c*^	79	1283	1	9	*aphA6*, *sul2*, *aadA1*, *dfrA1*, *strAB*, *cmlB1*, *oxa23*, *bla*_TEM_	JAHICN000000000
UC20804	109	1637	3	9	*aphA6*, *sul2*, *aacC2*, *aadA1* (2×), *dfrA1* (2×), *strAB*, *aadB*, *oxa58* (2×), *catB2*	CP076807–CP076811
UC21421	109	1637	3	9	*aadA*, *dfrA1*, *aadB*, *oxa58*, *catB2*	JAHICM000000000
UC22612	109	1637	3	9	*aphA6*, *sul2*, *aacC2*, *aadA*, *dfrA1*, *strAB*, *aadB*, *oxa58*, *catB2*	JAHICL000000000
UC23844	109	1637	3	9	*sul2*, *aacC2*, *aadA*, *dfrA1*, *strAB*, *aadB*, *oxa58*, *catB2*	JAHICK000000000
UC24803	109	1637	3	9	*aphA6*, *sul2*, *aacC2*, *aadA*, *dfrA1*, *strAB*, *aadB*, *oxa58*, *catB2*	JAHICJ000000000
UC25167^*d*^	109	1637	3	9	*aphA6*, *sul2*, *aacC2*, *aadA*, *dfrA1*, *strAB*, *aadB*, *oxa58*, *catB2*	JAHICI000000000
UC25224	109	1637	3	9	*aphA6*, *sul2*, *aacC2*, *aadA*, *dfrA1*, *strAB*, *aadB*, *oxa58*, *catB2*	JAHICH000000000
UC25235^*d*^	109	1637	3	9	*aphA6*, *sul2*, *aacC2*, *aadA*, *dfrA1*, *strAB*, *aadB*, *oxa58*, *catB2*	JAHICG000000000
UC25271	109	1637	3	9	*aphA6*, *sul2*, *aacC2*, *aadA*, *dfrA1*, *strAB*, *aadB*, *oxa58*, *catB2*	JAHICF000000000
UC25560	109	1637	3	9	*aadA*, *dfrA1*, *aadB*, *oxa58*, *catB2*	JAHICE000000000
UC20520	162	235	1	19	*aphA6*, *oxa23*, *mph-msr*(E)	JAHICD000000000
UC20976	162	235	1	19	*aphA6*, *oxa23*	JAHICC000000000
UC21311	162	235	1	19	*aphA6*, *aadA*, *oxa23*, *sul1*, *mph-msr*(E), *aadA2*, *armA*, *dfrA12*	JAHICB000000000
UC21742	162	235	1	19	*aphA6*, *sul2*, *aadA*, *oxa23*, *aadB*, *floR*	JAHICA000000000
UC24371	162	235	1	19	*aphA6*, *oxa23*	CP076804–CP076806
UC25604	162	235	1	19	*oxa23*	CP076801–CP076803

a ST^IP^, ST according to the Institut Pasteur MLST scheme (*cpn60-fusA-gltA-pyrG-recA-rplB-rpoB*).

b ST^OX^, ST according to the Oxford MLST scheme (*gltA-gyrB-gdhB-recA-cpn60-gpi-rpoD*).

c Isolates recovered from the same patient.

d Isolates recovered from the same patient.

**FIG 1:**
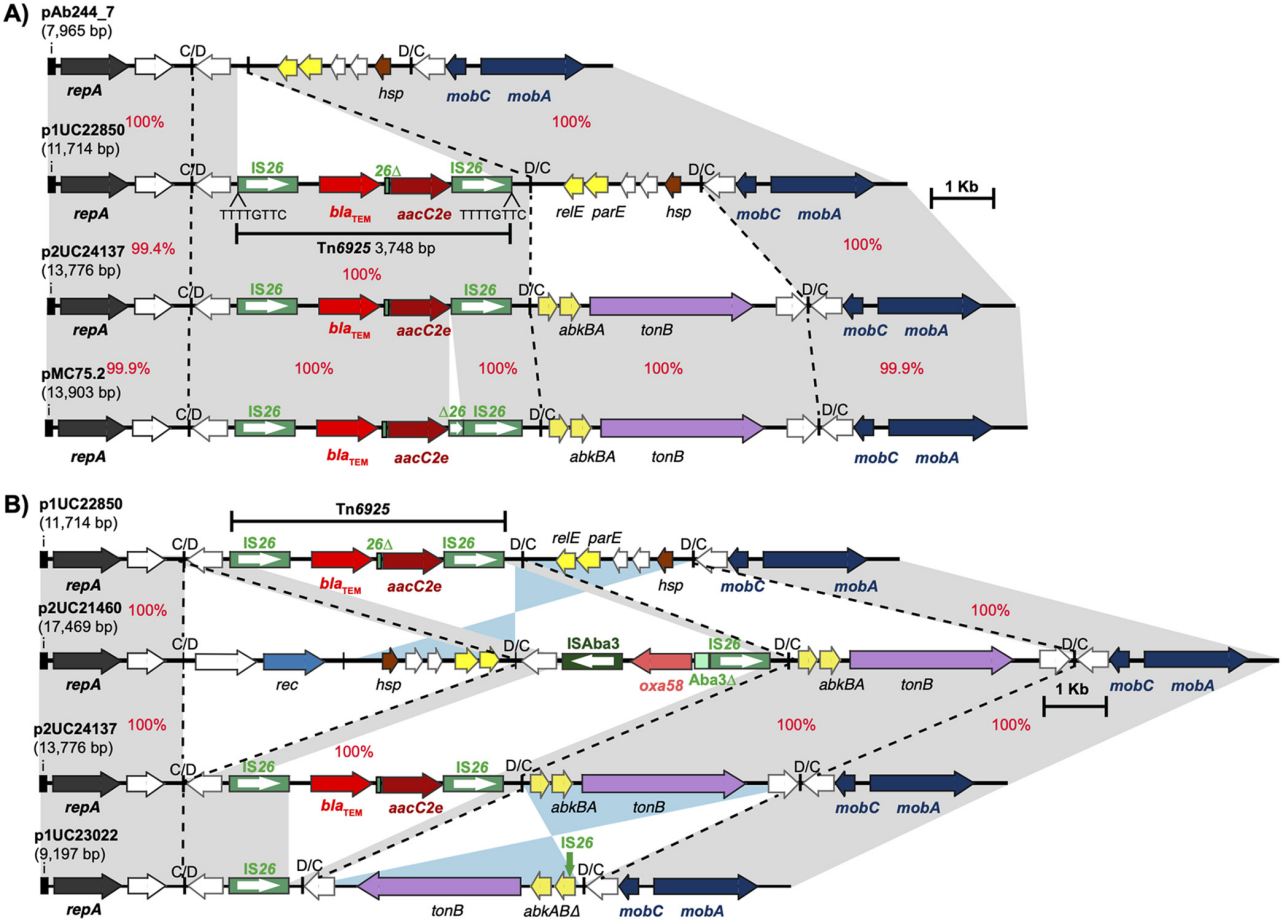
Comparison of small plasmids carrying Tn*6925* and *oxa58* found in Chilean strains. Central black lines show plasmid backbones, and small vertical lines indicate p*dif* sites. C/D and D/C are XerC/XerD binding sites. Plasmid names are shown on the left, with their sizes in base pairs in parentheses. Horizontal arrows show the extent and orientation of genes, and boxed white arrows indicate insertion sequences (ISs). Regions with significant homologies are shown using shades of gray, with red numbers indicating their DNA identities. pAb244_7, p1UC22850, p2UC24137, pMC75.2, p2UC21460, and p1UC23022 were drawn to scale from the data under GenBank accession numbers MG520098, CP076821 and CP076822, CP076817 to CP076820, MK531541, CP076814 to CP076816, and CP076812 and CP076813, respectively.

In UC22850, the *sul1*, *tet*(A), and *catA1* genes were found in a novel AbaR-type resistance island variant that includes two internal deletions mediated by IS*26*-2 (of Tn*6020*) removing parts of Tn*1* (on the left) and extending to the middle of open reading frame 4 (ORF4) (on the right). AbaR-type antibiotic resistance islands are often found in the chromosomal *comM* gene (17), and here, this novel AbaR-type variant was named AbaR36. The *bla*_TEM_ and *aacC2* resistance genes, which were separated by a remnant of IS*26* (61 bp), were found in a 3,748-bp IS*26*-bounded novel composite transposon, which was named Tn*6925* (Fig. 1). Tn*6925* is in an 11,714-bp plasmid, which was named p1UC22850 (GenBank accession number CP076822). p1UC22850 encodes a putative replication initiation protein (belonging to the Rep_3 family [Pfam01051]). Its closest match is *repAci4* (GenBank accession number GU978998), with 90.7% DNA identity. It also encodes the MobAC mobilization proteins, suggesting that it might be a mobilizable plasmid. To date, several A. baumannii plasmids have been shown to include specific modules, called p*dif* modules, flanked by inversely oriented binding sites for the XerCD recombinases, known as p*dif* sites (18, 19). p*dif* sites are often 28 bp and consist of two 11-bp inversely oriented binding sites separated by a 6-bp spacer. Analysis of p1UC22850 showed that Tn*6925* is inserted into a p*dif* module containing an additional reading frame encoding a hypothetical protein. p1UC22850 is identical to a 7,965-bp plasmid (pAb244_7 [GenBank accession number MG520098]), except that pAb244_7 does not contain Tn*6925* (Fig. 1). Hence, p1UC22850 appears to have diverged from pAb244_7 by the acquisition of Tn*6925*. Notably, pAb244_7 is carried by A. baumannii strain Ab244, which was isolated in Argentina. Antibiotic resistance genes were also found to be in a similar genomic context in the other two GC1 genomes determined here (UC22863 and UC23742).

Phylogenetic analysis of the core genomes of UC22850, UC22863, and UC23742 and a set of 17 GC1 strains known to represent different lineages within GC1 (20) showed that all three Chilean strains are tightly clustered (see Fig. S1 in the supplemental material). This suggests that the Chilean GC1 strains examined here belong to a lineage separate from those found on other continents.

### Strains belonging to the ST15 complex represent a diverse group.

**FIG 2:**
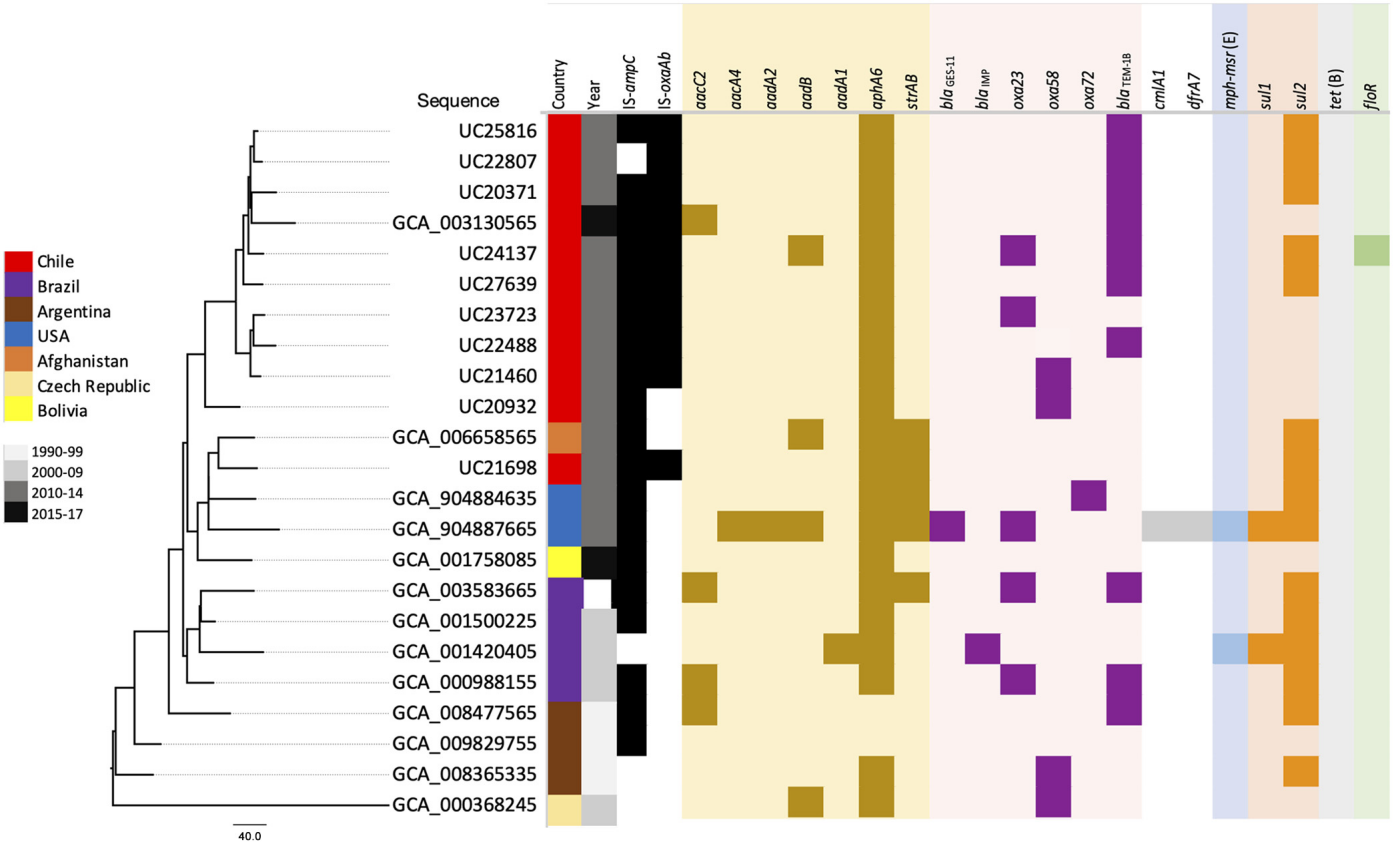
Phylogenetic tree of ST15/ST318 strains sequenced in this study and those publicly available in GenBank. Columns indicate the country of isolation, the year of isolation, the presence and absence of IS*Aba1* upstream of the intrinsic *ampC* and *oxaAb* genes, as well as the presence and absence of antibiotic resistance genes.

The group of strains belonging to the ST15 complex included 8 ST15 and 2 ST318 strains (Tables 1 and 2). ST318 is a single-locus variant of ST15 as it differs only in the *rpoB* gene. All 10 strains belonging to the ST15/ST318 group include OCL7, with all but 2, UC20932 and UC21698, carrying KL22 (Table 2). UC20932 and UC21698 include KL9. All 10 strains but 1, UC22807, included a copy of IS*Aba1* upstream of their chromosomal *ampC* genes, accounting for their resistance to third-generation cephalosporins (e.g., ceftazidime) (Fig. 2). A copy of IS*Aba1* was also found upstream of the intrinsic *oxaAb* gene (encoding OXA-219), contributing to their resistance to carbapenems (Fig. 2). All 10 strains (8 ST15 and 2 ST318 isolates) included the *aphA6* amikacin resistance gene in Tn*aphA6* found in a novel chromosomal location (present at position 3258404 in the sequence under GenBank accession number CP076817).

To resolve the context of their resistance regions, representatives of ST15 (UC24137) and ST318 (UC21460) were also selected, and their genomes were completed using a hybrid short- and long-read data approach.

The completed genome of UC24137 carries three plasmids, p1UC24137 (99,399 bp [GenBank accession number CP076818]), p2UC24137 (13,776 bp [GenBank accession number CP076819]), and p3UC24137 (9,543 bp [GenBank accession number CP076820]). p1UC24137 is a 99,399-bp plasmid encoding the putative RepAci6 replication initiation protein that belongs to the RepPriCT_1 family (Pfam03090). p1UC24137, which encodes RepAci6, carries 4 antibiotic resistance genes, the *aadB* tobramycin, *floR* florfenicol, *sul2* sulfonamide, and *oxa23* carbapenem resistance genes. The *oxa23* gene was found in a Tn*2008*::IS*Aba12* structure inserted into the plasmid backbone, while *aadB*, *floR*, and *sul2* were part of an ~29-kb resistance region. The *aadB* gene cassette was found in a 1.6-kb truncated class 1 integron bounded by an IS*26* and IS*Aba52* and with its entire 3′ conserved sequence (CS) and gene cassettes missing. The *floR-sul2* region is similar to the one in pJ9-3 characterized previously (GenBank accession number CP041590.1), which is an unrelated A. baumannii plasmid. The *sul2* and *strAB* genes in other ST15 strains were also predicted to be in a similar region in a RepAci6 plasmid.

The complete genome sequence of UC21460 (ST318) includes the chromosome (3,910,207 bp [GenBank accession number CP076814]) and two plasmids, p1UC21460 (55,507 bp [GenBank accession number CP076815]) and p2UC21460 (17,469 bp [GenBank accession number CP076816]). UC21460 contains two *aphA6* copies, both in Tn*aphA6*. One Tn*aphA6* copy is in the same chromosomal location as that in other ST15/ST318 strains (at base 2803909 in the sequence under GenBank accession number CP076814), and the second copy is in yet another novel chromosomal position (base 3761582 in the sequence under GenBank accession number CP076814). UC21460 also contains an *oxa58* gene that accounts for the carbapenem resistance phenotype observed. The *oxa58* gene was found in an IS*Aba3*-*oxa58-*IS*Aba3*Δ-IS*26* structure in p2UC21460 (Fig. 1B). p2UC21460 is a 17,469-bp plasmid related to p2UC24137 and p1UC22850 as it shares the same backbone (encoding Rep and MobAC) with them. p2UC21460 also shares the *tonB* p*dif* module with p2UC24137 and shares the *relE-parE* toxin-antitoxin module with p1UC22850. p2UC21460 differs from p2UC24137 and p1UC22850 by the acquisition of a new p*dif* module (encoding a recombinase) (Fig. 1B) that contains the IS*Aba3*-*oxa58-*IS*Aba3*Δ-IS*26* structure. Interestingly, the IS*Aba3*-*oxa58-*IS*Aba3*Δ-IS*26* structure was in a p*dif* module precisely where Tn*6925* would be in p2UC24137 and p1UC22850 (Fig. 1B). In fact, the IS*Aba3*-*oxa58-*IS*Aba3*Δ-IS*26* structure replaces Tn*6925*, presumably due to an IS*26*-mediated event. An identical p*dif* module containing the IS*Aba3*-*oxa58-*IS*Aba3*Δ-IS*26* structure was found in several A. baumannii and non-*baumannii* Acinetobacter species plasmids (GenBank accession numbers JQ241789.1, CP026086.2, CP026749.2, CP033558.1, and CP033547.1), indicating the spread of *oxa58* due to the acquisition of this p*dif* module. Interestingly, a plasmid identical to p2UC21460 was also found in UC20932, which is another member of the ST15 group that we sequenced here (Tables 1 and 2).

To put Chilean ST15/ST318 genomes into a global context, we constructed a core-genome single nucleotide polymorphism (cgSNP) phylogenetic tree of Chilean strains as well as those publicly available (13 genomes) as of December 2021. All but three ST15 genomes were from isolates recovered in South America, indicating their significance in the region. Ten (*n* = 10) Chilean strains, including 9 from this study and 1 reported previously (strain Ab3_Ch [GenBank assembly accession number GCA_003130565]), formed a large cluster, confirming their relatedness (Fig. 2). Only one isolate was not part of this cluster (UC21698) and was closely related to an Afghan genome, which indicates that UC21698 might have been an imported strain given that it includes *strAB*, the same as in the Afghan genome, while none of the other ST15/ST318 genomes studied here contained these genes. Notably, all but two genomes, from Argentina (GenBank assembly accession numbers GCA_008477565 and GCA_009829755), contained the chromosomal Tn*aphA6* (Fig. 2). The lack of Tn*aphA6* and both genomes being from strains recovered in the 1990s suggest that these two strains are likely to represent ancestral ST15 strains. Of the two strains, Ab825 (GenBank accession numbers GCA_009829755 and NTFR00000000) lacks any antibiotic resistance gene, making it the ancestral strain of all ST15 strains. Therefore, other strains may have diverged from it by antibiotic resistance gene acquisition events involving several transposons and plasmids.

### Chilean ST79:ST1283:KL1:OCL9 isolates carrying a copy of Tn*7*::Tn*3* represent a distinct homogeneous group within the South American ST79 clade.

Five ST79 strains were sequenced and analyzed in this study. Analysis of the genomic features of these strains revealed a homogeneous group. Members of this group all include the KL9 and OCL1 surface polysaccharides and belong to ST1283 according to the Oxford multilocus sequence typing (MLST) scheme (ST1283^OX^), making them ST79:ST1283:KL1:OCL9. All strains included a copy of IS*Aba1* upstream of the intrinsic chromosomal *ampC* gene, accounting for their resistance to third-generation cephalosporins. IS*Aba1* could not be found upstream of the chromosomal *oxaAb* gene in any of the ST79 genomes. All ST79 isolates carried the *aphA6*, *sul2*, *aadA1*, *dfrA1*, *strAB*, *cmlB1*, *bla*_TEM_, and *oxa23* resistance genes, indicating no variation in their resistance gene repertoire.

To study the context of antibiotic resistance genes, the complete genome sequence of UC23022 (representing the ST79 group) was determined. UC23022 (GenBank accession number CP076812) contains a cryptic plasmid called p1UC23022 (9,197 bp [GenBank accession number CP076813]). Interestingly, all antibiotic resistance genes were found to be in the chromosome of UC23022. The *aphA6* amikacin resistance gene is in Tn*aphA6* in a novel chromosomal position (at base 851056 in the sequence under GenBank accession number CP076812). The *oxa23* carbapenem resistance gene was in Tn*2008*, also in a novel chromosomal position (position 2887095).

**FIG 3:**
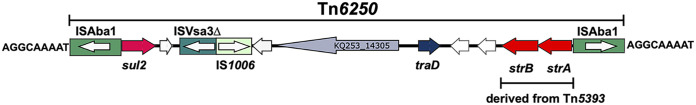
Genetic structure of Tn*6250*. Horizontal arrows illustrate genes, with antibiotic resistance genes in red. Green boxes show insertion sequences (ISs). The AGGCAAAAT sequence flanking Tn*6250* shows the 9-bp duplication of the target site generated by the insertion of Tn*6250*, a property of IS*Aba1*.

The *sul2* sulfonamide resistance gene was also found in the chromosome in Tn*6250* (bases 2939628 to 2952551 in the sequence under GenBank accession number CP007712). Tn*6250* was previously described in the chromosome of the A. baumannii strain LAC-4 (21). Tn*6250* is a composite 12,924-bp transposon made of a 10,564-bp central fragment bounded by two IS*Aba1* copies. In addition to *sul2*, Tn*6250* also contains a region derived from Tn*5393* that includes the streptomycin-spectinomycin resistance genes *strAB* (Fig. 3). In UC23022, Tn*6250* is flanked by a 9-bp (AGGCAAAAT) duplication of the target site, indicating the insertion of a Tn in this position.

**FIG 4:**
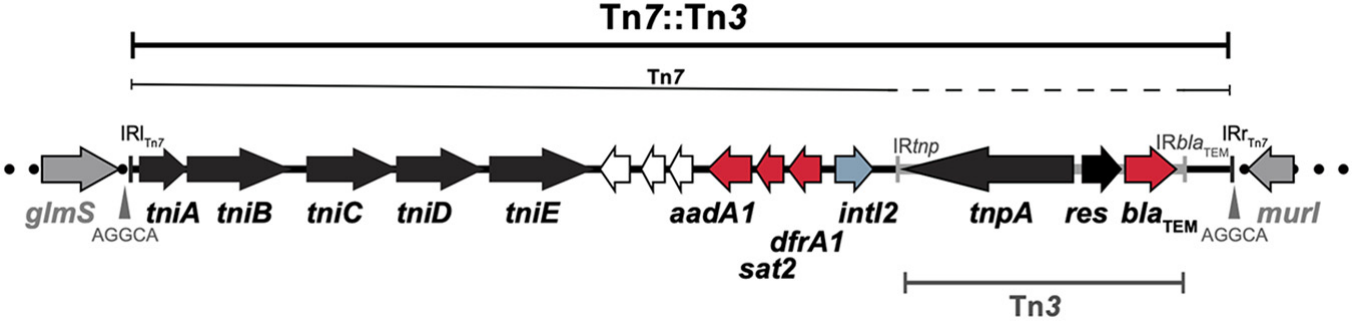
Genetic structure of Tn*7*::Tn*3* found in ST15 strains. The central thick horizontal black line indicates the backbone of Tn*7*::Tn*3*, and its flanking dotted lines indicate the chromosomal sequence. Horizontal arrows show the extent and orientation of genes, with antibiotic resistance genes in red, transposition genes in black, and chromosomal genes in gray. White arrows are open reading frames encoding hypothetical proteins. Inverted repeats (IRs) of Tn*7* and Tn*3* are shown using small vertical bars. The AGGCA sequence flanking Tn*7*::Tn*3* indicates the 5-bp duplication of the target site generated upon the insertion of Tn*7*.

The remaining antibiotic resistance genes, *aadA1*, *sat2*, *dfrA1*, and *bla*_TEM_, were part of a 19,020-bp Tn*7*::Tn*3* structure (bases 3121026 to 3140045 in the sequence under GenBank accession number CP007712) found downstream of the *glmS* gene, which is the preferred target site for Tn*7* (Fig. 4). Analysis of the *bla*_TEM_ region showed that it is part of a complete copy of Tn*3*, which has been inserted downstream of the *intI2* gene in the Tn*7* backbone (Fig. 4). The entire Tn*7*::Tn*3* structure was surrounded by the expected 5-bp duplication of the target site (AGGCA), indicating the transposition of Tn*7*. The Tn*3* copy is identical to the original Tn*3* sequence in the Escherichia coli plasmid R1 (GenBank accession number HM749966). An identical copy of Tn*7*::Tn*3* was also found in the GenBank nonredundant database in A. baumannii strain MRSN15313 (GenBank accession number CP033869.1), which also belongs to ST79. The ST79 isolates were collected from unrelated cases in three different hospitals between July 2011 and September 2012. Interestingly, the genomic contexts of all antibiotic resistance genes were identical in all other Chilean members of the ST79 group that we studied here (Table 2).

**FIG 5:**
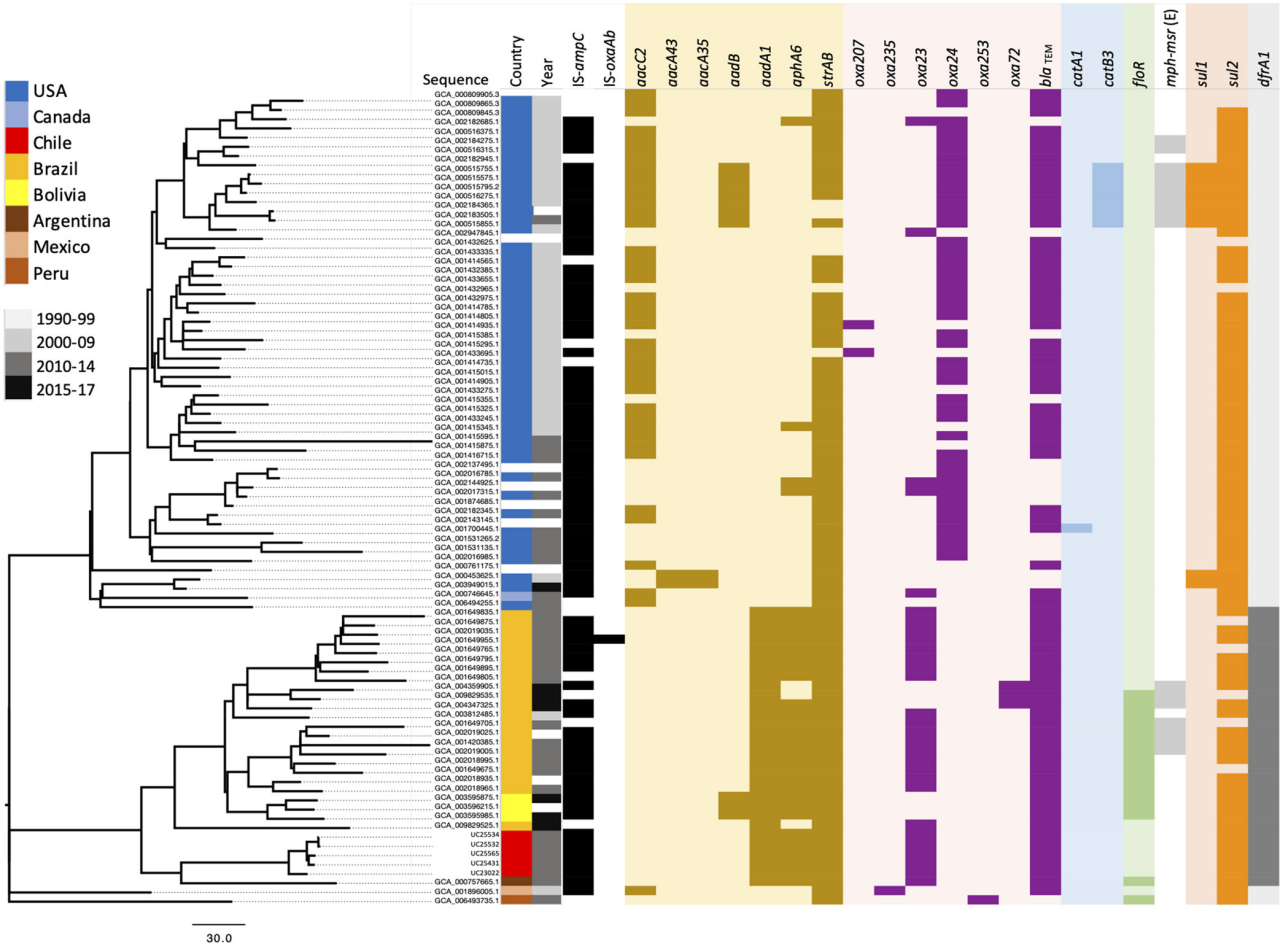
Phylogenetic tree of ST79 strains examined in this study and those found in GenBank. Columns indicate the country of isolation, the year of isolation, the presence and absence of IS*Aba1* upstream of the intrinsic *ampC* and *oxaAb* genes, as well as the presence and absence of antibiotic resistance genes.

As of December 2021, there were 88 ST79 genomes available in GenBank. Phylogenetic analysis of our strains combined with those found in GenBank yet again revealed that all Chilean strains were tightly clustered. Notably, a major split in the SNP phylogenetic tree separates all South American isolates from those recovered in North America, indicating two major clades in the ST79 population structure (Fig. 5). Interestingly, most North American isolates were recovered in the 1990s, suggesting that the two major clades have evolved separately over the last 2 decades. This is supported by the analysis of the context of antibiotic resistance genes. For example, both clades share few antibiotic resistance genes. Most South American isolates carry the Tn*7*::Tn*3* structure that we identified in Chilean isolates (represented by the presence of *aadA1* and *dfrA1*), while most North American isolates carry a variant of the small plasmids that carry the *aacC2* gene and were characterized here (e.g., p2UC24137 and p1UC22850) (Fig. 1).

### ST109:ST1637:KL3:OCL9 strains carry two copies of Tn*7* carrying *aadA1*, *sat2*, and *dfrA1*.

Ten isolates of ST109 according to the Institut Pasteur MLST scheme (ST109^IP^) were sequenced and analyzed here. Notably, the two ST109 strains were recovered from outpatients. All 10 genomes belong to ST1637^OX^ and also included KL9 and OCL3, making them ST109:ST1637:KL3:OCL9. All genomes included a copy of IS*Aba1* upstream of the intrinsic *ampC* gene, accounting for their resistance to third-generation cephalosporins. No IS was found upstream of the intrinsic *oxaAb* gene. While there are differences, ST109^IP^ strains share several antibiotic resistance genes, including *aphA6*, *sul2*, *aadA1*, *dfrA1*, *strAB*, *aadB*, *oxa58*, and *catB2*. Upon analysis of short-read assemblies and to resolve the context of resistance genes, one representative strain (UC20804), with the largest number of antibiotic resistance genes, was selected, and its genome was completed using a hybrid short- and long-read assembly approach.

The complete genome sequence of UC20804 consisted of the chromosome (GenBank accession number CP076807) and four plasmids, p1UC20804 (65,432 bp), p2UC20804 (17,469 bp), p3UC20804 (8,229 bp), and p4UC20804 (6,829 bp) (GenBank accession numbers CP076808, CP076809, CP076810, and CP076811, respectively). UC20804 was found to include two copies of Tn*7*, which contains the *aadA1*, *sat2*, and *dfrA1* resistance genes. One copy was found in its standard location (downstream of *glmS*), and the second copy was found elsewhere in a novel chromosomal position. The remainder of the resistance genes were found in different plasmids, e.g., p1UC20804.

p1UC20804 is a 65-kb plasmid that carries two novel putative replication initiation proteins belonging to the Rep_3 family (Pfam01051). p1UC20804 carries a copy of Tn*aphA6* (containing the *aphA6* amikacin resistance gene), *sul2* (sulfonamide resistance), *strAB* (streptomycin and spectinomycin resistance), and *aacC2* (aminoglycoside resistance). p2UC20804 carries the *oxa58* carbapenem resistance gene and was found to be identical to p2UC21460 (Fig. 1), indicating the circulation of this plasmid and its variants in the strains that we analyzed here. Analysis of the draft genomes of the remainder of the ST109 strains showed that all of the shared antibiotic resistance genes were in the same genomic context, confirming that they are closely related.

ST109^IP^ is a relatively rare sequence type. As of December 2021, there was only a single ST109 genome available in GenBank; hence, phylogenetic analysis was not carried out on this genome.

### ST162:ST235:KL1:OC19 strains represent an emerging sequence type.

All six ST162 genomes analyzed here were found to be ST162:ST235:KL1:OC19. They all included IS*Aba1* upstream of the intrinsic *ampC* gene, while no IS was detected preceding the intrinsic *oxaAb* gene. ST162 also represents a rare sequence type with only a single representative genome in GenBank. Our eBURST analysis suggests that ST162 appears to be an emerging sequence type (Fig. S2) given its low frequency and that it diverges from ST374. The ST162 strains analyzed here contain a diverse set of antibiotic resistance genes. All of these strains carried *oxa23*, which was found in plasmids encoding the RepAci6 putative replication initiation protein described above. All strains but one (UC25604) contained Tn*aphA6* in their chromosomes. Given the novelty of ST162, here, we completed the genome sequences of two ST162:ST235:KL1:OC19 strains, UC24371 and UC25604. Each genome contained a RepAci6 plasmid, p1UC25604 (88,371 bp) or p1UC24371 (105,056 bp) (GenBank accession numbers CP076802 and CP076805, respectively), that carried *oxa23* in Tn*2008*. Using draft genome assemblies, we also predicted a similar RepAci6 plasmid in all other ST162 strains. While all ST162 strains were resistant to carbapenems and third-generation cephalosporins, they contained a limited and diverse repertoire of antibiotic resistance genes.

## DISCUSSION

Treatment of infections caused by carbapenem-resistant A. baumannii (CR*Ab*) strains has become a global challenge given the wide distribution of clones that are resistant to most commercially available antibiotics (2, 4). The spread of CR*Ab* is due mainly to the dissemination of a few global clones, with GC2 strains being substantially the most widespread clones, followed by members of the GC1 group (6, 22). However, this trend varies in different geographical regions (6, 22, 23). In the United States and most countries, including European countries, Australia, and China, most CR*Ab* strains belong to the ST2 clonal complex (6), while other carbapenem resistance sequence types are also emerging, e.g., ST85 in the Middle East (10), ST499 in the United States (11), and ST25 globally (6). However, while CR*Ab* is prevalent in South America, GC1 strains are rare, and GC2 strains are almost nonexistent (12–14). Instead, strains of other carbapenem-resistant sequence types such as ST15 and ST79 are prevalent (3, 12–14, 24, 25). However, the genetic context of antibiotic resistance genes in the dominant South American clones and, in particular, Chilean isolates had not been examined in detail.

Here, consistent with the results of previous studies, our analysis of 34 carbapenem-resistant isolates recovered from a Chilean reference hospital and its satellite clinics/hospitals revealed no GC2 strains, whereas only three GC1 strains were found. Instead, and again consistent with the results of previous studies (3, 12–14, 24, 25), several sequence types, namely, ST15/ST318, ST79, ST109, and ST162, were identified. ST15 and ST79 are well established in many South American countries (20). ST109 and ST162 have also been reported in Brazil (26) and Chile (14). ST162 strains were initially described in South Korea (21), followed by a few reports from Brazil although at lower abundances than ST15 and ST79, which are now known as major South American sequence types (27, 28). ST162 strains are often carbapenem resistant and associated with *oxa23* (21, 28).

The overarching finding of this study is that while a wide range of known and novel mobile genetic elements (MGEs) are involved in the acquisition of antibiotic resistance genes in Chilean strains (sequence type-specific mechanisms), several mobile genetic elements also play a crucial role in the spread of important resistance genes, such as aminoglycoside and carbapenem resistance genes, across different sequence types. For instance, Tn*aphA6* is an important transposon that spreads the *aphA6* amikacin resistance gene in all sequence types found in this study. Plasmids encoding the RepAci6 putative initiation proteins also play a crucial role in the dissemination of the *oxa23* carbapenem resistance gene (often in Tn*2008* or its IS-interrupted variants) as well as several other genes such as the *sul2* sulfonamide resistance gene and the *strAB* streptomycin-spectinomycin resistance genes. Most notably, variants of small plasmids related to p2UC24137, p1UC22850, p2UC20804, or p2UC21460, which we characterized in detail here, are also responsible for the spread of aminoglycoside and carbapenem resistance genes (e.g., *aacC2* and *oxa58*). Our findings suggest that these small plasmids, which are made of several p*dif* modules that are widespread in small plasmids of A. baumannii, e.g., the *tonB* or *relE-parE* modules, encode a novel Rep_3 family replication initiation protein. Our comparative analysis showed that the *aacC2* gene, along with *bla*_TEM_, is in Tn*6925* (Fig. 1), inserted into a p*dif* module encoding a hypothetical protein. Finding the IS*Aba3*-*oxa58*-IS*Aba3*Δ-IS*26* structure in p2UC21460, precisely where Tn*6925* is present in other related plasmid variants (such as p2UC24137 or p1UC22850), and the cryptic variant of this plasmid type in our set (p1UC23022) (Fig. 1B) suggests that the acquisition of these important resistance genes had occurred by IS*26*-mediated events, as previously described (29, 30). Moreover, we found that Tn*7* and its variant Tn*7*::Tn*3* have played important roles in the acquisition of several resistance genes (*aadA1*, *sat2*, *dfrA1*, and *bla*_TEM_) in ST79 and ST109 strains.

The core-genome single nucleotide polymorphism (cgSNP) phylogenetic analysis performed here confirmed that all Chilean strains of each sequence type (ST1, ST15, and ST79) are closely related and diverged from related strains recovered from different countries. Within each ST group, Chilean strains could be differentiated from each other by only dozens of SNPs; however, given that information on patient transmission between hospital wards, admission, or discharge, etc., was not available, it was not possible to use this limited number of cgSNPs to infer transmission within hospital wards.

Notably, phylogenetic analysis of the ST79 strains revealed two distinct North and South American clades. Analysis of the acquired antibiotic resistance genes and the mobile genetic elements associated with them indicates that this clone is established in both North and South America, and while they share an ancestor (given their shared MGEs), they have also evolved differently in each region, acquiring several differences in their resistance gene repertoires. For example, the acquisition of the Tn*7*::Tn*3* structure by South American ST79 strains clearly differentiates them from other North American ST79 members.

This work studied details of the genetic context of antibiotic resistance genes in a set of carbapenem-resistant A. baumannii strains recovered from two Chilean hospitals and revealed a complex evolutionary picture of antibiotic resistance gene acquisition events via multiple routes involving several mobile genetic elements. Although this study may not reflect the real prevalence of sequence types circulating in the country, given that the samples came from the same hospital network, it draws a detailed picture of a fraction of the strains circulating in the country. Additional future work will also be needed to track the evolution of resistance over time and how circulating clones respond to possible changes (or maintenance) of antibiotic administration policies in the region. This study adds 34 CR*Ab* genomes to the publicly available pool of GenBank genome sequence data. This will be important to better understand the global epidemiology of antibiotic resistance in the World Health Organization’s number one antibiotic resistance research priority pathogen, namely, CR*Ab*.

## MATERIALS AND METHODS

### Bacterial strain collection.

From a total of 164 clinical isolates of Acinetobacter baumannii obtained at the Catholic University Hospital and the San Carlos Catholic University Clinic between 2010 and 2013, 34 isolates that were resistant to carbapenems (meropenem and/or imipenem) were studied. Only one isolate per patient was considered, except for isolates UC25431 and UC25565, which came from the same patient, as well as UC25167 and UC25235, which were recovered from a second patient. In both cases, the second isolates were recovered approximately 4 weeks after the first strains were recovered, indicating persistent infections. The sources of isolation included 6 isolates from blood cultures, 8 from respiratory specimens, 6 from wounds, 7 from tissue, 1 from leg bone, 2 from urine, 4 from secretion liquids, and 1 from a catheter tip (Table 1). The median age of the patients was 61 years. Inpatients were hospitalized in wards of Clinical Hospital UC, named A, B, and C in Table 1.

### Antibiotic resistance profile.

The susceptibilities to seven categories of antibiotics were determined. Specifically, susceptibility to amikacin, gentamicin, meropenem, imipenem, ampicillin-sulbactam, cefoperazone-sulbactam, ceftazidime, and ciprofloxacin (all antibiotics were obtained from Sigma) was determined using the standard agar dilution method (31) with breakpoints suggested by the Clinical and Laboratory Standards Institute (CLSI) (26). Briefly, bacterial suspensions of each strain were adjusted to a 0.5 McFarland standard and dispensed into the seeding-tray wells of a Cathra replicator system (32 wells). Mueller-Hinton agar plates supplemented with various concentrations of antibiotics were seeded using 1-mm pins. Susceptibility to colistin was determined by measuring MICs using the standard agar dilution method as previously described (31) and interpreted according to CLSI guidelines (26). Susceptibility to tigecycline and colistin was determined using the standard broth microdilution assay.

### Genomic DNA extraction, sequencing, and assembly.

Isolates were grown overnight on LB agar, and single colonies were obtained and suspended in 5 mL of LB broth in a Falcon tube and incubated overnight at 37°C. One milliliter of the liquid culture was used for DNA extraction using the Qiagen DNeasy blood and tissue kit according to the manufacturer’s instructions. Library preparation was performed using the Hackflex protocol (32), and sequencing was performed on an Illumina MiSeq system at the University of Technology Sydney, Ultimo, sequencing facility. The short reads were assembled using Shovill (https://github.com/tseemann/shovill). After an initial analysis of the genomes, seven isolates were selected as representatives of sequence types (STs) and sequenced using long-read technology on an Oxford Nanopore GridION flow cell at the Garvan Institute of Medical Research, Sydney, Australia. Filtlong was used to filter long reads with poor quality and those that were <1 kb long. Reads were then trimmed using the Porechop (v.0.2.3) program (33), using default parameters. The high-quality Illumina and GridION reads were assembled *de novo* using a hybrid assembly approach using the Unicycler program (v.0.4.7) (34).

### Genome annotation and characterization.

Assembled sequences were annotated using Prokka (35) and deposited into the NCBI GenBank database. The assembled contigs were screened for antimicrobial resistance genes using ABRicate (https://github.com/tseemann/abricate), with the ResFinder reference database (36). MLST of the assemblies was performed using MLST software (https://github.com/tseemann/mlst) according to the Institut Pasteur (which uses *cpn60-fusA-gltA-pyrG-recA-rplB-rpoB*) and Oxford (which uses *gltA-gyrB-gdhB-recA-cpn60-gpi-rpoD*) schemes. eBURST sequence type cluster analysis was performed only for the emerging ST162 using the PubMLST database (https://pubmlst.org/organisms/acinetobacter-baumannii) and BIGSdb (Bacterial Isolate Genome Sequence Database) (available at https://bigsdb.readthedocs.io/en/latest/). BLAST (37) was used to characterize antibiotic resistance regions and associated mobile genetic elements using known reference sequences as the queries. BLAST was also used to determine the presence or absence of any IS upstream of the intrinsic *oxaAb* and *ampC* genes. Insertion sequences were identified using the ISFinder database (https://www-is.biotoul.fr). Surface polysaccharide loci K (capsule) and OC (outer core) were typed using Kaptive (38). SnapGene software (v.6.0.5) was used to manually annotate the regions of interest and draw figures to scale using the Illustrator program (v.26.2.1).

### Phylogenetic analysis.

Separate phylogenetic trees were constructed for each sequence type found in the study samples (ST1, ST15, ST79, ST109, and ST162). Since ST1 is a well-characterized sequence type in the literature, known references (*n* = 17) were used to reconstruct the phylogenies. For the remaining sequence types, references were obtained from the NCBI genome assembly database; all A. baumannii genomes were downloaded (>5,000 genomes as of October 2021). The sequence types of the reference sequences were determined using MLST software (https://github.com/tseemann/mlst). All reference sequences from ST15 and ST79 were used to construct the phylogenies. The phylogenies of ST162 and ST109 were not estimated because only one reference sequence was available. Maximum likelihood phylogenetic trees were constructed from a core-genome alignment of the A. baumannii genomes studied here. The study and reference sequences for each sequence type were aligned with Snippy v.4.6.0 using sequence assembly ASM211692v1 (GenBank assembly accession number GCA_002116925.1) as the reference for the assembly. The phylogenetic trees were constructed using the Snippy-aligned sequences using Gubbins v.2.4.1 (39). The Gubbins algorithm estimates tree phylogenies using SNPs (single nucleotide polymorphisms) in areas not suggestive of horizontal sequence transfer (recombination). High-quality core-genome SNPs identified by Gubbins that differentiated the genomes were extracted and used to construct phylogenetic trees. Final maximum likelihood phylogenetic trees were inferred from the resulting alignment using RAxML (v.8) with the generalized time-reversible (GTR) gamma model of nucleotide substitution, as previously described (40). Trees were visualized using Figtree (https://github.com/rambaut/figtree/releases). Similar to the approach used to determine the antibiotic resistance genes in the study sequences, all reference assemblies were screened using ABRicate software (https://github.com/tseemann/abricate) with the ResFinder database (36).

### Data availability.

The genome sequence data (including complete and draft genome assemblies) for all strains determined in this study have been deposited in the GenBank/EMBL/DDBJ database and are publicly available under GenBank BioProject accession number PRJNA731249.

## References

[1] Chng KR, Li C, Bertrand D, Ng AHQ, Kwah JS, Low HM, Tong C, Natrajan M, Zhang MH, Xu L, Ko KKK, Ho EXP, Av-Shalom TV, Teo JWP, Khor CC, MetaSUB Consortium, Chen SL, Mason CE, Ng OT, Marimuthu K, Ang B, Nagarajan N. 2020. Cartography of opportunistic pathogens and antibiotic resistance genes in a tertiary hospital environment. Nat Med 26:941–951. doi:10.1038/s41591-020-0894-4. 32514171 PMC7303012

[2] Abbott I, Cerqueira GM, Bhuiyan S, Peleg AY. 2013. Carbapenem resistance in *Acinetobacter baumannii*: laboratory challenges, mechanistic insights and therapeutic strategies. Expert Rev Anti Infect Ther 11:395–409. doi:10.1586/eri.13.21. 23566149

[3] Camargo CH, Cunha MPV, de Barcellos TAF, Bueno MS, Bertani AMDJ, dos Santos CA, Nagamori FO, Takagi EH, Chimara E, de Carvalho E, Tiba-Casas MR. 2020. Genomic and phenotypic characterisation of antimicrobial resistance in carbapenem-resistant *Acinetobacter baumannii* hyperendemic clones CC1, CC15, CC79 and CC25. Int J Antimicrob Agents 56:106195. doi:10.1016/j.ijantimicag.2020.106195. 33045346

[4] Nordmann P, Poirel L. 2019. Epidemiology and diagnostics of carbapenem resistance in Gram-negative bacteria. Clin Infect Dis 69:S521–S528. doi:10.1093/cid/ciz824. 31724045 PMC6853758

[5] Tacconelli E, Carrara E, Savoldi A, Harbarth S, Mendelson M, Monnet DL, Pulcini C, Kahlmeter G, Kluytmans J, Carmeli Y, Ouellette M, Outterson K, Patel J, Cavaleri M, Cox EM, Houchens CR, Grayson ML, Hansen P, Singh N, Theuretzbacher U, Magrini N, WHO Pathogens Priority List Working Group. 2018. Discovery, research, and development of new antibiotics: the WHO priority list of antibiotic-resistant bacteria and tuberculosis. Lancet Infect Dis 18:318–327. doi:10.1016/S1473-3099(17)30753-3. 29276051

[6] Hamidian M, Nigro SJ. 2019. Emergence, molecular mechanisms and global spread of carbapenem-resistant *Acinetobacter baumannii*. Microb Genom 5:e000306. doi:10.1099/mgen.0.000306. 31599224 PMC6861865

[7] Nigro SJ, Hall RM. 2016. Structure and context of *Acinetobacter* transposons carrying the *oxa23* carbapenemase gene. J Antimicrob Chemother 71:1135–1147. doi:10.1093/jac/dkv440. 26755496

[8] Nigro SJ, Hall RM. 2018. Does the intrinsic *oxaAb* (*bla*OXA-51-like) gene of *Acinetobacter baumannii* confer resistance to carbapenems when activated by ISAba1? J Antimicrob Chemother 73:3518–3520. doi:10.1093/jac/dky334. 30124881

[9] da Silva KE, Maciel WG, Croda J, Cayô R, Ramos AC, de Sales RO, Kurihara MNL, Vasconcelos NG, Gales AC, Simionatto S. 2018. A high mortality rate associated with multidrug-resistant *Acinetobacter baumannii* ST79 and ST25 carrying OXA-23 in a Brazilian intensive care unit. PLoS One 13:e0209367. doi:10.1371/journal.pone.0209367. 30592758 PMC6310363

[10] Mann R, Rafei R, Gunawan C, Harmer CJ, Hamidian M. 2022. Variants of Tn*6924*, a novel Tn*7* family transposon carrying the *bla*_NDM_ metallo-β-lactamase and 14 copies of the *aphA6* amikacin resistance genes found in *Acinetobacter baumannii*. Microbiol Spectr 10:e01745-21. doi:10.1128/spectrum.01745-21. 35019774 PMC8754128

[11] Iovleva A, Mustapha MM, Griffith MP, Komarow L, Luterbach C, Evans DR, Cober E, Richter SS, Rydell K, Arias CA, Jacob JT, Salata RA, Satlin MJ, Wong D, Bonomo RA, van Duin D, Cooper VS, Van Tyne D, Doi Y. 2022. Carbapenem-resistant *Acinetobacter baumannii* in U.S. hospitals: diversification of circulating lineages and antimicrobial resistance. mBio 13:e02759-21. doi:10.1128/mbio.02759-21. 35311529 PMC9040734

[12] Cifuentes S, Moura Q, Cardoso B, Esposito F, Cerdeira L, Álvarez E, Barrera E, Opazo-Capurro A, Gonzalez-Rocha G, Lincopan N. 2020. Genomic features of a carbapenem-resistant OXA-219-positive *Acinetobacter baumannii* of international ST15 (CC15) from a patient with community-onset urinary tract infection in Chilean Patagonia. J Glob Antimicrob Resist 22:756–758. doi:10.1016/j.jgar.2020.07.011. 32712382

[13] Nodari CS, Cayô R, Streling AP, Lei F, Wille J, Almeida MS, de Paula AI, Pignatari ACC, Seifert H, Higgins PG, Gales AC. 2020. Genomic analysis of carbapenem-resistant *Acinetobacter baumannii* isolates belonging to major endemic clones in South America. Front Microbiol 11:584603. doi:10.3389/fmicb.2020.584603. 33329450 PMC7734285

[14] Opazo-Capurro A, San Martín I, Quezada-Aguiluz M, Morales-León F, Domínguez-Yévenes M, Lima CA, Esposito F, Cerdeira L, Bello-Toledo H, Lincopan N, González-Rocha G. 2019. Evolutionary dynamics of carbapenem-resistant *Acinetobacter baumannii* circulating in Chilean hospitals. Infect Genet Evol 73:93–97. doi:10.1016/j.meegid.2019.04.022. 31029791

[15] Subsecretaria de Redes Asistenciales, Ministerio de Salud. 2020. Informe de Vigilancia de IAAS 2018, ORD C37/M2565. Ministerio de Salud, Santiago, Chile.

[16] Magiorakos AP, Srinivasan A, Carey RB, Carmeli Y, Falagas ME, Giske CG, Harbarth S, Hindler JF, Kahlmeter G, Olsson-Liljequist B, Paterson DL, Rice LB, Stelling J, Struelens MJ, Vatopoulos A, Weber JT, Monnet DL. 2012. Multidrug-resistant, extensively drug-resistant and pandrug-resistant bacteria: an international expert proposal for interim standard definitions for acquired resistance. Clin Microbiol Infect 18:268–281. doi:10.1111/j.1469-0691.2011.03570.x. 21793988

[17] Hamidian M, Hall RM. 2018. The AbaR antibiotic resistance islands found in *Acinetobacter baumannii* global clone 1—structure, origin and evolution. Drug Resist Updat 41:26–39. doi:10.1016/j.drup.2018.10.003. 30472242

[18] Blackwell GA, Hall RM. 2017. The *tet39* determinant and the *msrE*-*mphE* genes in *Acinetobacter* plasmids are each part of discrete modules flanked by inversely oriented p*dif* (XerC-XerD) sites. Antimicrob Agents Chemother 61:e00780-17. doi:10.1128/AAC.00780-17. 28533235 PMC5527579

[19] Hamidian M, Blasco L, Tillman LN, To J, Tomas M, Myers GSA. 2020. Analysis of complete genome sequence of *Acinetobacter baumannii* strain ATCC 19606 reveals novel mobile genetic elements and novel prophage. Microorganisms 8:1851. doi:10.3390/microorganisms8121851. 33255319 PMC7760358

[20] Cameranesi MM, Paganini J, Limansky AS, Moran-Barrio J, Salcedo SP, Viale AM, Repizo GD. 2020. Acquisition of plasmids conferring carbapenem and aminoglycoside resistance and loss of surface-exposed macromolecule structures as strategies for the adaptation of *Acinetobacter baumannii* CC104^O^/CC15^P^ strains to the clinical setting. Microb Genom 6:mgen000360. doi:10.1099/mgen.0.000360. 32213259 PMC7643966

[21] Lee Y, Bae IK, Kim J, Jeong SH, Lee K. 2012. Dissemination of ceftazidime-resistant *Acinetobacter baumannii* clonal complex 92 in Korea. J Appl Microbiol 112:1207–1211. doi:10.1111/j.1365-2672.2012.05283.x. 22404202

[22] Douraghi M, Kenyon JJ, Aris P, Asadian M, Ghourchian S, Hamidian M. 2020. Accumulation of antibiotic resistance genes in carbapenem-resistant *Acinetobacter baumannii* isolates belonging to lineage 2, global clone 1, from outbreaks in 2012–2013 at a Tehran burns hospital. mSphere 5:e00164-20. doi:10.1128/mSphere.00164-20. 32269158 PMC7142300

[23] Higgins PG, Hagen RM, Kreikemeyer B, Warnke P, Podbielski A, Frickmann H, Loderstädt U. 2021. Molecular epidemiology of carbapenem-resistant *Acinetobacter baumannii* isolates from northern Africa and the Middle East. Antibiotics (Basel) 10:291. doi:10.3390/antibiotics10030291. 33799540 PMC8002098

[24] Correa A, Del Campo R, Escandón-Vargas K, Perenguez M, Rodríguez-Baños M, Hernández-Gómez C, Pallares C, Perez F, Arias CA, Cantón R, Villegas MV. 2018. Distinct genetic diversity of carbapenem-resistant *Acinetobacter baumannii* from Colombian hospitals. Microb Drug Resist 24:48–54. doi:10.1089/mdr.2016.0190. 28570118 PMC5802270

[25] Levy-Blitchtein S, Roca I, Plasencia-Rebata S, Vicente-Taboada W, Velásquez-Pomar J, Muñoz L, Moreno-Morales J, Pons MJ, Del Valle-Mendoza J, Vila J. 2018. Emergence and spread of carbapenem-resistant *Acinetobacter baumannii* international clones II and III in Lima, Peru. Emerg Microbes Infect 7:119. doi:10.1038/s41426-018-0127-9. 29970918 PMC6030224

[26] Clinical and Laboratory Standards Institute. 2019. Performance standards for antimicrobial susceptibility testing; 29th informational supplement. Clinical and Laboratory Standards Institute, Wayne, PA.

[27] Chagas TP, Carvalho KR, de Oliveira Santos IC, Carvalho-Assef AP, Asensi MD. 2014. Characterization of carbapenem-resistant *Acinetobacter baumannii* in Brazil (2008-2011): countrywide spread of OXA-23-producing clones (CC15 and CC79). Diagn Microbiol Infect Dis 79:468–472. doi:10.1016/j.diagmicrobio.2014.03.006. 24880823

[28] de Azevedo FKSF, Dutra V, Nakazato L, Mello CM, Pepato MA, de Sousa ATHI, Takahara DT, Hahn RC, Souto FJD. 2019. Molecular epidemiology of multidrug-resistant *Acinetobacter baumannii* infection in two hospitals in central Brazil: the role of ST730 and ST162 in clinical outcomes. J Med Microbiol 68:31–40. doi:10.1099/jmm.0.000853. 30516469

[29] Harmer CJ, Hall RM. 2017. Targeted conservative formation of cointegrates between two DNA molecules containing IS*26* occurs via strand exchange at either IS end. Mol Microbiol 106:409–418. doi:10.1111/mmi.13774. 28833671

[30] Harmer CJ, Moran RA, Hall RM. 2014. Movement of IS*26*-associated antibiotic resistance genes occurs via a translocatable unit that includes a single IS*26* and preferentially inserts adjacent to another IS*26*. mBio 5:e01801-14. doi:10.1128/mBio.01801-14. 25293759 PMC4196232

[31] Andrews JM. 2001. Determination of minimum inhibitory concentrations. J Antimicrob Chemother 48(Suppl 1):5–16. doi:10.1093/jac/48.suppl_1.5. 11420333

[32] Gaio D, Anantanawat K, To J, Liu M, Monahan L, Darling AE. 2022. Hackflex: low-cost, high-throughput, Illumina Nextera Flex library construction. Microb Genom 8:e000744. doi:10.1099/mgen.0.000744. PMC8914357 35014949

[33] Wick RR, Judd LM, Gorrie CL, Holt KE. 2017. Completing bacterial genome assemblies with multiplex MinION sequencing. Microb Genom 3:e000132. doi:10.1099/mgen.0.000132. 29177090 PMC5695209

[34] Wick RR, Judd LM, Gorrie CL, Holt KE. 2017. Unicycler: resolving bacterial genome assemblies from short and long sequencing reads. PLoS Comput Biol 13:e1005595. doi:10.1371/journal.pcbi.1005595. 28594827 PMC5481147

[35] Seemann T. 2014. Prokka: rapid prokaryotic genome annotation. Bioinformatics 30:2068–2069. doi:10.1093/bioinformatics/btu153. 24642063

[36] Bortolaia V, Kaas RS, Ruppe E, Roberts MC, Schwarz S, Cattoir V, Philippon A, Allesoe RL, Rebelo AR, Florensa AF, Fagelhauer L, Chakraborty T, Neumann B, Werner G, Bender JK, Stingl K, Nguyen M, Coppens J, Xavier BB, Malhotra-Kumar S, Westh H, Pinholt M, Anjum MF, Duggett NA, Kempf I, Nykäsenoja S, Olkkola S, Wieczorek K, Amaro A, Clemente L, Mossong J, Losch S, Ragimbeau C, Lund O, Aarestrup FM. 2020. ResFinder 4.0 for predictions of phenotypes from genotypes. J Antimicrob Chemother 75:3491–3500. doi:10.1093/jac/dkaa345. 32780112 PMC7662176

[37] Altschul SF, Gish W, Miller W, Myers EW, Lipman DJ. 1990. Basic local alignment search tool. J Mol Biol 215:403–410. doi:10.1016/S0022-2836(05)80360-2. 2231712

[38] Wyres KL, Cahill SM, Holt KE, Hall RM, Kenyon JJ. 2020. Identification of *Acinetobacter baumannii* loci for capsular polysaccharide (KL) and lipooligosaccharide outer core (OCL) synthesis in genome assemblies using curated reference databases compatible with Kaptive. Microb Genom 6:e000339. doi:10.1099/mgen.0.000339. 32118530 PMC7200062

[39] Croucher NJ, Page AJ, Connor TR, Delaney AJ, Keane JA, Bentley SD, Parkhill J, Harris SR. 2015. Rapid phylogenetic analysis of large samples of recombinant bacterial whole genome sequences using Gubbins. Nucleic Acids Res 43:e15. doi:10.1093/nar/gku1196. 25414349 PMC4330336

[40] Kozlov AM, Darriba D, Flouri T, Morel B, Stamatakis A. 2019. RAxML-NG: a fast, scalable and user-friendly tool for maximum likelihood phylogenetic inference. Bioinformatics 35:4453–4455. doi:10.1093/bioinformatics/btz305. 31070718 PMC6821337

