# Supplementary material for: Genomic Analysis of Carbapenem-Resistant Acinetobacter baumannii Strains Recovered from Chilean Hospitals Reveals Lineages Specific to South America and Multiple Routes for Acquisition of Antibiotic Resistance Genes
Source: Microbiol Spectr. 2022 Sep 26;10(5):e02463-22. doi: 10.1128/spectrum.02463-22 (PMC9602995; doi:10.1128/spectrum.02463-22)
Supplement: Supplemental file 1 — Fig. S1 and S2. Download spectrum.02463-22-s0001.pdf, PDF file, 0.2 MB [file spectrum.02463-22-s0001.pdf]

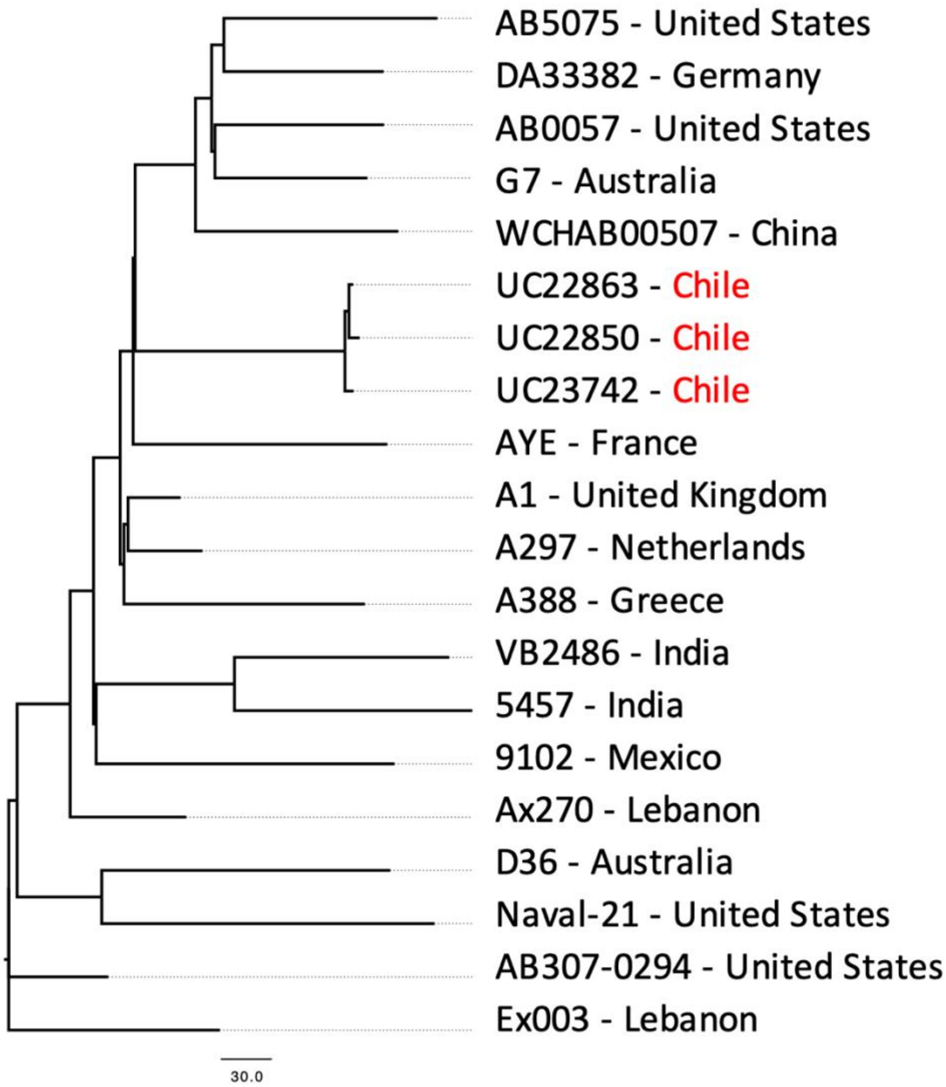

Figure S1. Phylogenetic tree of three ST1 strains examined in this studied and known lineage reference of the global GC1. The Chilean sequences collected in this study are separately grouped into a specific lineage.

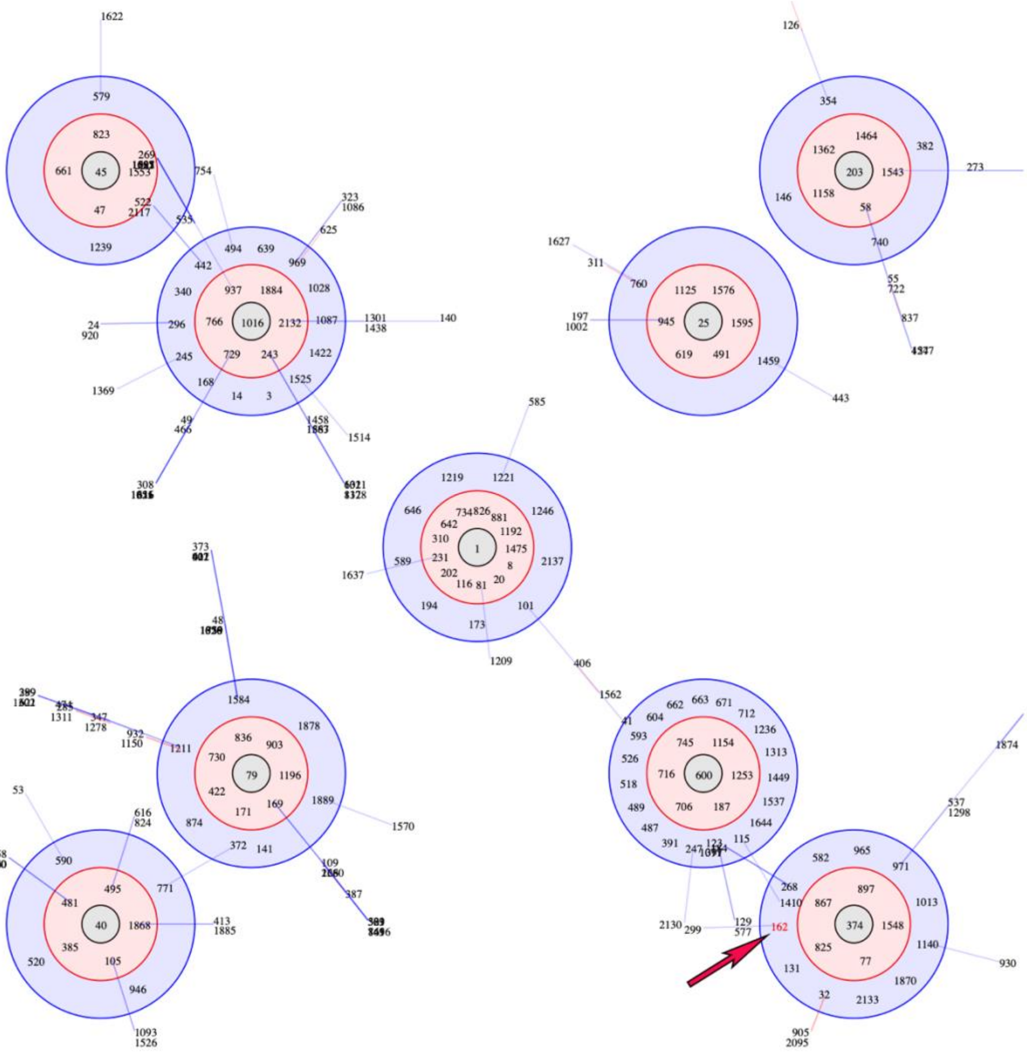

Figure S2. eBURST showing the position of ST162 relative its most related sequence types.

The red arrow indicates ST162.
